# Acupuncture therapy on chronic fatigue syndrome based on radar plot: A protocol for an overview of systematic reviews

**DOI:** 10.1097/MD.0000000000024572

**Published:** 2021-04-09

**Authors:** Lijie Tang, Taijun Jiang, Feng-Ya ZHu, ZHengkang Liu, Xi Wu

**Affiliations:** a School of Acu-Mox and Tuina; b Acupuncture Clinical Research Center, Chengdu University of Traditional Chinese Medicine, Chengdu 610075, Sichuan, China.

**Keywords:** acupuncture, AMSTAR-2, chronic fatigue syndrome, multiple evaluations, overview, PRISMA, radar plot

## Abstract

**Background:**

Chronic fatigue syndrome (CFS) is a debilitating chronic disease of unknown etiology that is recognized by the World Health Organization (WHO) and the United States Center for Disease Control and Prevention (US CDC) as a disorder of the brain. CFS affects 1% (17–24 million people) of the world's population and is a major and costly public health problem. In traditional Chinese medicine (TCM), acupuncture can achieve a certain effect in the treatment of chronic fatigue syndrome, but evidence-based medicine is controversial. This protocol aims to multi-evaluate the literature quality and evidence quality of the current systematic reviews (SRs)/meta-analyses (MAs) of acupuncture treatment for chronic fatigue syndrome, and provide intuitive and reliable evidence synthesis and decision-making basis for clinical treatment.

**Methods:**

Eight databases will be searched from their inception to 1 June, 2020: the Chinese Biomedical Literature Database (CBM), China Science and Technology Journal Database (VIP), China National Knowledge Infrastructure (CNKI), WanFang Database (WF), Web of Science, Embase, PubMed, and Cochrane Library. Published systematic reviews that were reported in Chinese or English, and the included studies were randomized controlled clinical trials (RCTs) for acupuncture in people with CFS will be included. Reviews selection, data extraction and management, and assessment of the study quality will be completed independently by 2 or more reviewers. The quality of evidence, methodological quality, and reporting quality will be evaluated by using the Grading of Recommendations Assessment, Development, and Evaluation (GRADE), A Measurement Tool to Assessment of Multiple Systematic Reviews-2 (AMSTAR-2), Preferred Reporting Items for Systematic Reviews and Meta-analyses (PRISMA), and Adobe Illustrator Creative Cloud (Adobe Illustrator CC) will be used to draw and optimize the radar plot.

**Results:**

The article in this overview will be submitted for publication in a peer-reviewed journal.

**Conclusion:**

We hope to collect evidence from accessible and useful systematic reviews of acupuncture treatment for chronic fatigue syndrome, to provide visual and scientific decision-making methods for more clinical practice and medical research.

**Systematic Review Registration:**

INPLASY 202060052.

## Abstract 2

Supplemental Digital Content is available in the text

## 1 Introduction

Chronic fatigue syndrome is a heterogeneous and disabling clinical disease.^[1–4]^ The core symptom is difficult to relieve without a clear cause, fatigue lasting at least 6 months, and is accompanied by sleep abnormalities, cognitive impairment, and orthostatic intolerance.^[3]^ Symptoms of tolerance, pain, flu-like physical or mental activity abnormalities are related to the central nervous system, autonomic nervous system, digestive system, endocrine metabolism, immune regulation, and other systems.^[4,5]^ International research on the etiology, pathogenesis, and treatment of chronic fatigue syndrome (CFS) involves more than 20 commonly used case definition standards.^[2]^ The prevalence of CFS/ME ranges from 0.1% to 7.62%, affecting different ages, races, and socioeconomic groups.^[6]^ Some studies have shown that approximately 3 to 4 times as many women as men present the symptoms.^[7,8]^ According to existing literature, as early as 1999, CFS has been reported to be more common than lung cancer and AIDS^[9]^ and as widespread as rheumatoid arthritis.^[10]^ The latest review of the ME/CFS, reported by the American Institute of Medicine (IOM), estimated that 836,000 to 2.5 million Americans suffered from ME/CFS, and each family lost approximately $20,000 in personal income per annum and suffered from CFS. The unemployment rate of the people is between 35% and 69%,^[11]^ and it consumes approximately US$17 to 24 billion in financial costs each year.^[12]^ Some studies have estimated that the complete cure rate of CFS is not higher than 5%, and most of the disease lasts for several years, even decades. In April 2019, the National Institutes of Health (NIH) sponsored the “Accelerate Research on ME/CFS,” meeting that it was necessary to increase the research funding of CFS. Obviously, CFS seriously affects life and work, bringing patients and society a heavy financial burden.^[3]^

According to the existing literature, there have been many systematic reviews (SRs)/meta-analysis (MA) studies on the treatment of CFS,^[13–19]^ but these studies cannot determine the best plan, and there are no clear drugs or complementary/alternative therapies for the cure of CFS. At present, the US Food and Drug Administration has not approved treatments for CFS,^[4]^ but there are already some measures that can control or alleviate some symptoms, including drug intervention, physical therapy, psychological counseling, dietary therapy, and complementary and alternative medicine therapies. Acupuncture is one of the recommended alternative therapies for CFS.^[9]^ It is widely used in China to relieve fatigue, sleep abnormalities, cognitive dysfunction, and pain in CFS.^[20–28]^ With the increase in clinical research and mechanism research on acupuncture treatment of CFS, the SRs/MAs related to it also increase accordingly. Some SRs/meta-analyses studies have shown that acupuncture (hand acupuncture, warm acupuncture, electroacupuncture) treatment of CFS is superior to the control group in improving clinical effectiveness, alleviating fatigue, improving sleep, and fewer adverse reactions. While some studies have shown that due to the lack of high-quality research, the effectiveness and safety of acupuncture treatment of CFS cannot be clarified.^[29]^ Some studies suggest that acupuncture treatment of CFS can improve inflammatory factors, and hormone levels require follow-up observation or further expansion of the study.^[21,30]^ In addition, some studies indicate that acupuncture cannot effectively improve fatigue.^[31]^

High-quality SRs/MAs are a prerequisite for providing reliable evidence for clinical practice. However, few SRs/MAs of CFS related to acupuncture treatment are currently conducted at home and abroad. This study will use Grading of Recommendations Assessment, Development, and Evaluation (GRADE) to assess the quality of evidence from the publication year, design type, Assessment of Multiple Systematic Reviews-2 (AMSTAR-2) methodological quality score, Preferred Reporting Items for Systematic Reviews and Meta-analyses (PRISMA) report quality score, homogeneity, and publication bias risk. Multiple evaluations of acupuncture treatment CFS SRs/MAs of literature quality, using Adobe Illustrator Creative Cloud (Adobe Illustrator CC) draws and optimizes radar charts to achieve visual evaluation and provide a visual decision basis for clinical decision-making.

## 2 Objectives

The objectives are as following:

(1) Comprehensively evaluate the quality of evidence, methodological quality, and report quality of acupuncture treatment CFS, and assess what aspects need to continue to improve.
(2) Multi-evaluation of the literature quality of CFS SRs/MAs of acupuncture treatment using radar charts to provide a visual decision-making basis for the clinic.

## 3 Methods

### 3.1 Registration of the review

The protocol will be written in accordance with the Preferred Reporting Items for Overview of SRs (PRIO-harms).^[32]^ This protocol has been registered with the International Platform of Registered Systematic Review and Meta-Analyses Protocols (INPLASY), registration number: INPLASY202060052, DOI number: 10.3776/inplasy 2020.6.0052 (https://inplasy.com/inplasy-2020-6-0052/).

### 3.2 Inclusion and exclusion criteria

Following the recommendations of the Cochrane Handbook for SRs of Interventions,^[33]^ the included studies that meet the PICOS (Population, Intervention, Comparison, Outcome, and Study) strategy, will be considered for inclusion in this overview. Duplicate publications, protocols, meetings, abstracts, non-full text, expert experience, animal experiment, literature on non-acupuncture treatment of chronic fatigue syndrome, and other reviews will be excluded.

#### 3.2.1 Type of studies

Published SRs reported in Chinese or English, and the included studies were randomized controlled clinical trials (RCTs) for acupuncture in people with CFS.

#### 3.2.2 Type of participants

We will include patients with CFS. The course of disease, age, sex, and race for patients with CFS will be unlimited.

#### 3.2.3 Type of interventions

Acupuncture, electroacupuncture, fire needle, auricular acupuncture, catgut embedding, auricular therapy, acupressure, acupoint injection, or any combination of the above.

#### 3.2.4 Types of comparisons

Placebo, sham acupuncture, drugs, psychotherapy, or other conventional treatments, including no treatment.

#### 3.2.5 Types of outcomes

##### 3.2.5.1 Primary outcomes

(1) Total effective rate,
(2) Fatigue scale like Chalder's Fatigue Scale (CFQ) and Fatigue Scale-14 (FS-14).

##### 3.2.5.2 Secondary outcomes

(1) 36-Item Short-Form Health Survey (SF-36),
(2) Hamilton Anxiety Scale,
(3) Hamilton Depression Scale,
(4) Visual Analogue Scale.

### 3.3 Data collection

#### 3.3.1 Search methods for identification of reviews

**Figure 1:**
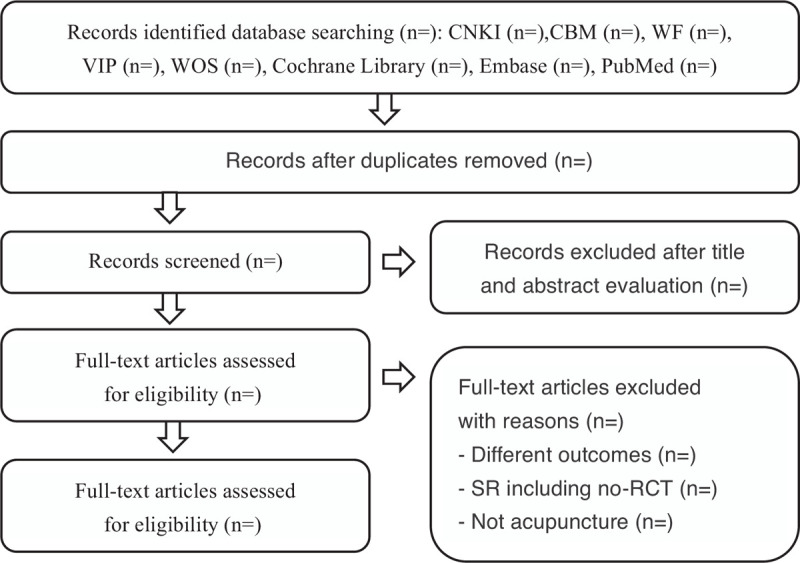
Flow chart of literature search and study selection process of MAs/SRs of CFS. CFS = chronic fatigue syndrome, MAs = meta-analyses, SRs = systematic reviews.

We will comprehensively search 8 electronic databases, including Cochrane Library, EMBASE, Web of Science, and PubMed and 4 Chinese electronic databases (Wanfang Database, China National Knowledge Infrastructure [CNKI], Chinese Scientific Journals Database [VIP], and Chinese Biomedical Database [CBM]) from their inception to 1 June, 2020. The language of publication is limited to Chinese or English. The search strategy for each database used as following: (“Chronic Fatigue Syndrome” OR “Fatigue Syndrome, Chronic” OR “Myalgic Encephalomyelitis”) AND (“acupuncture” OR “acupuncture therapy” OR “acupoint” OR “Zhenjiu” OR “Zhenci” OR “scalp-acupuncture” OR “auricular acupuncture” OR “catgut embedding” OR “auricular therapy” OR “acupressure”) AND (“Meta analyses” OR “Systematic review”), etc. The PRISMA flow chart of studies for MAs/SRs is shown in Figure 1 and the search strategy is given in Supplemental Digital Content (Appendix 1, http://links.lww.com/MD/F624).

#### 3.3.2 Selection of reviews

Search results of bibliographies will be first imported into NoteExpress 3.2.0, and duplicate publications will be removed through NoteExpress 3.2.0. Two reviewers (ZFY and LZK) independently read the literature. According to the title and abstract, the studies that did not meet the inclusion requirements will be excluded, and those that matched the requirements will be downloaded and read in full text to determine whether they met the inclusion criteria. In case of disagreements, a third party (Wu Xi) will be consulted to assist in study selection. In cases of missing data, the author will be contacted.

#### 3.3.3 Data extraction and management

Two researchers (TLJ and JTJ) will independently extract the data according to the inclusion and exclusion criteria. In the process, the extracted data will be submitted to a third party (WX) for inspection and verification. In case of disagreement, a decision shall be made after discussion with the third party.

Excel 2019 will be used to build the information extraction table, named SRs/MAs literature quality assessment information collection table for acupuncture therapy of chronic fatigue syndrome. According to the pre-designed data, 2 tables for extracting data were created. Table 1 includes the basic characteristics of the included studies: first author, year of publication, nationality of the first author, number of studies included, sample size, intervention measures, control measures, assessment tool for risk of bias, primary outcome, secondary outcome. Table 2 lists the multi-evaluation and rank number of the 6 dimensions of this study: first author, year of publication, type of study, AMSTAR-2, PRISMA, homogeneity, publication bias, and rank average score.

**Table 1:** Basic characteristics of the included studies.

					Intervention			Outcome	
First author	Year	Nationality	Number	Sample size	T	C	Assessment tool for risk of bias	Primary	Secondary
									

**Table 2:** Multivariate evaluation and rank number of 6 dimensions of the included studies.

First author	Year	Type	AMSTAR-2	PRISMA	Homogeneity	Publication bias	Rank average score
							

PRISMA = Preferred Reporting Items for Systematic Reviews and Meta-analyses.

#### 3.3.4 Critical appraisal of included reviews

The following literature evaluation processes were performed independently by two researchers. If there are disputed opinions, all the researchers will discuss at the meeting until a unified consensus is reached. The inter-rater agreement between the two researchers will be measured using the Kappa statistic.^[34]^

### 3.4 Data synthesis

We will make a narrative description of the SRs/MAs of the included studies and create tables to detail the contents and results of the above lectures. In addition, we will integrate these articles and provide comprehensive treatment effects for all SRs, including the scores of the primary and secondary outcome indicators. For each outcome indicator, we will conduct a subgroup analysis, comparing acupuncture or acupuncture combined with other therapies with drugs. If necessary, the results will be meta-analyzed with RevMan5.3 software. The summary effect size was estimated using the mean difference (MD) with 95% confidence intervals (CI) for continuous outcomes.

### 3.5 Subgroup analysis

If feasible, subgroup analysis will be performed according to gender, different types of acupuncture methods, severity of condition, or intervention period.

### 3.6 Radar chart drawing principles

A radar plot is a multivariate analysis tool that can use quantitative indicators to better reflect qualitative problems. The petal length of the radar plot represents the conditions of each variable, and the overall area of the petals shows the general condition. According to the medical statistics grade data processing method, the scores of each evaluation item are converted into ranks. The number of the literatures is the highest rank value, and the rest of the evaluation items are based on the total number of documents as the highest score, and the highest score is assigned to the top ranked. The six-dimensional index average is the rank average score.

### 3.7 Evaluation of the quality of the included reviews

#### 3.7.1 Assessment of methodological quality of included reviews

A measurement tool to assess multiple SRs-2 (AMSTAR-2)^[35]^ was used to evaluate the methodological quality of each SR. Each item in the AMSTAR 2 scale was standardized and scored as 1 for correct use, 0 for unused or misused, with a full score of 11.

#### 3.7.2 Report quality of included reviews

Each included MA/SR was scored on a case-by-case basis concerning the PRISMA.^[36]^ Each item in the PRISMA scale had 1 point for proper use, 0.5 point for incomplete use, 0 point for unused or misuse, and a full score of 27.

#### 3.7.3 Quality of evidence in included reviews

The GRADE^[37]^ was used to evaluate the primary outcome indicators, including 5 downgrading factors of risk of bias, indirectness, inconsistency, imprecision, and publication bias, and 3 upgrading factors: large effect size, dose–effect relationship, and negative bias. For the research type included in the literature, the initial evidence quality of RCT alone is high quality, and the initial evidence quality of other types of literature is of medium quality. Then, the outcome indicators are evaluated one by one according to the above 5 degrading factors and 3 upgrading factors. Finally, the quality of the evidence is divided into 4 grades: high quality, medium quality, low quality, and very low quality from high to low. Evidence for each of the selected clinical outcomes in the table will be filled with the summary of estimated risk and 95% confidence intervals (Table 3).

**Table 3:** GRADE classification results.

				Degradation factors							
First author	Type	Outcomes	Confidence intervals (95%)	Outcome	Risk of bias	Inconsistency	Indirectness	Imprecision	Publication bias	Upgrade factors	Evidence quality
											

GRADE = Grading of Recommendations Assessment, Development, and Evaluation.

### 3.8 Homogeneity

*I*^2^ and Chi-square tests were used to evaluate the heterogeneity of the included trials. When more than half of the outcome indicators included in the literature have *P* ≥ .01 and *I*^2^ ≤ 50%, it indicates high homogeneity.

### 3.9 Published bias

The assessment of reporting bias was performed using the funnel chart and Egger's method.^[38,39]^ If the literature uses funnel charts or other methods to evaluate publication bias, the risk of publication bias is low. If you ignore publication bias, the risk of publication bias will be high.

## Author contributions

**Conceptualization:** Lijie Tang, TaiJun Jang, Xi Wu.

**Data curation:** Lijie Tang, Taijun Jiang, FengYa ZHu, ZHengKang Liu.

**Formal analysis:** Lijie Tang, TaiJun Jang.

**Funding acquisition:** Xi Wu.

**Investigation:** Lijie Tang.

**Methodology:** Lijie Tang.

**Project administration:** Xi Wu.

**Resources:** Lijie Tang, Xi Wu.

**Software:** Taijun Jiang.

**Supervision:** FengYa ZHu, ZHengKang Liu, Xi Wu.

**Validation:** Lijie Tang.

**Visualization:** Lijie Tang, Xi Wu.

**Writing – original draft:** Lijie Tang, TaiJun Jang.

**Writing – review & editing:** Lijie Tang, Xi Wu.
